# Magnetic Array‐Aided Visualizing PEMFC Degradation Heterogeneity

**DOI:** 10.1002/advs.202403631

**Published:** 2024-06-17

**Authors:** Yuning Sun, Lei Mao, Zhiyong Hu, Xiaoyu Zhang, Ranran Peng

**Affiliations:** ^1^ Department of Precision Machinery and Precision Instrumentation University of Science and Technology of China Hefei 230027 China; ^2^ Institute of Advanced Technology University of Science and Technology of China Hefei 230031 China; ^3^ Department of Materials Science and Engineering University of Science and Technology of China Hefei 230026 China

**Keywords:** degradation heterogeneity, magnetic array, proton exchange membrane fuel cell, visualization

## Abstract

Analyzing degradation heterogeneity of proton exchange membrane fuel cell (PEMFC) while maintaining high practicality is consistently challenging, primarily due to the destructive and costly nature of existing techniques relying on material characterization. In this work, a designed magnetic array integrating 16 sensors within 25 cm^2^ space is used for direct scanning and imaging of PEMFC performance heterogeneity during its degradation. Results are validated through degradation mechanism analysis and material characterization, confirming its potential in guiding the development of durable materials.

## Graphical abstract

Sensing PEMFC degradation heterogeneity with high practicality is consistently challenging. A designed magnetic array integrating 16 sensors within 25 cm^2^ space is easily‐implemented and non‐destructive for direct imaging of performance heterogeneity during PEMFC degradation, providing insights into structure disintegration and material fracture. The magnetic array is equally effective for other types of batteries such as lithium‐ion batteries, etc.

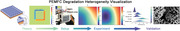

## 1 Introduction

PEMFC holds promise as the next‐generation energy source for various applications, boasting high energy conversion efficiency, rapid start‐up capability, and environmental friendliness.^[^ ^1^, ^2^, ^3^, ^4^ ^]^ Despite these advantages, PEMFC durability remains a significant obstacle, prompting interest in clarifying PEMFC degradation.^[^ ^5^, ^6^, ^7^ ^]^ Previous studies have revealed varying degradation rates across different zones on the membrane electrode assembly (MEA) surface,^[^ ^8^, ^9^, ^10^ ^]^ indicating in‐plane degradation heterogeneity within PEMFC, which is attributed to increased inconsistencies in material properties, and considered as the primary factor contributing to accelerated degradation and limited lifespan.^[^ ^11^, ^12^ ^]^ Therefore, monitoring and analyzing PEMFC degradation heterogeneity is crucial for understanding material fracture and structural disintegration during its lifetime.

In recent decades, various approaches, particularly material characterization tests, have been employed to analyze PEMFC failure modes and degradation mechanisms under different operating conditions, utilizing accelerated stress tests (AST).^[^ ^13^, ^14^ ^]^ Conventional material characterization techniques, such as scanning electron microscopy (SEM), transmission electron microscopy (TEM), X‐ray diffraction (XRD), etc., can evaluate MEA structure disintegration, but their destructive testing characteristics hinder continuous degradation heterogeneity analysis. More recently, advanced methods designed for continuous assessment, such as operando neutron imaging and operando X‐ray scattering tomography, have been introduced.^[^ ^15^, ^16^ ^]^ Nevertheless, these techniques are limited by specialized experimental scenarios that cannot be replicated in real‐life applications.

With PEMFC heterogeneous degradation, electrochemical reaction rates vary among MEA surface, resulting in diverse current rates.^[^ ^17^ ^]^ Consequently, sensing current distribution within MEA can be a viable way to characterize PEMFC degradation heterogeneity. Currently, segmented cells and printed circuit boards (PCBs) have been fabricated for intrusive measurements of current distribution within PEMFC.^[^ ^18^, ^19^ ^]^ However, as customized PEMFC structure is required for these methods, they are unsuitable for integration into commercial PEMFC systems. Building upon these challenges, alternative approaches have been proposed, focusing on collecting external excited magnetic field to indirectly evaluate PEMFC internal current distribution.^[^ ^20^, ^21^ ^]^ Unfortunately, most existing studies utilize single magnetic sensor, which cannot accurately reveal the distribution analysis.

In this study, a specific designed high‐integration magnetic array imaging device is used to sense magnetic field, thus enabling direct imaging of PEMFC degradation heterogeneity. Owing to innovative design for minimizing interference, magnetic array can integrate multiple sensors to directly deploy on PEMFC surface, and has a broader sensing range to provide more detailed information about magnetic field variation among PEMFC surface, fabricating more precise heterogeneity analysis. Results highlight magnetic array imaging device as a tool to provide continuous and non‐destructive material fracture and structural disintegration analysis.

## 2 Results and Discussion

### 2.1 PEMFC Degradation Heterogeneity Induced In‐Plane Current

**Figure 1:**
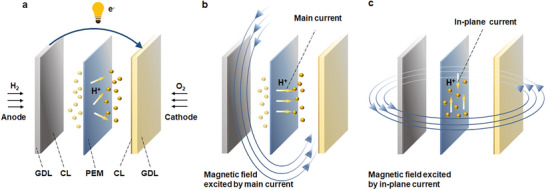
Current distributions and corresponding magnetic fields within PEMFC. a) Internal current; b) Main current and corresponding magnetic field; c) In‐plane current and corresponding magnetic field.

As aforementioned, PEMFC degradation heterogeneity introduces an uneven current distribution within proton exchange membrane (PEM) surface. According to Butler–Volmer equation, this inhomogeneous current distribution leads to potential difference, which results in in‐plane current flows.^[^ ^22^ ^]^ As depicted in **Figure** **1**a, internal current within PEMFC can be decomposed into the main current and in‐plane current, where the main current (Figure 1b) is formed by protons perpendicularly passing through PEM to achieve redox reaction and generate output voltage, while in‐plane current (Figure 1c) is parasitic loss and flows parallel to PEM surface.

**Figure 2:**
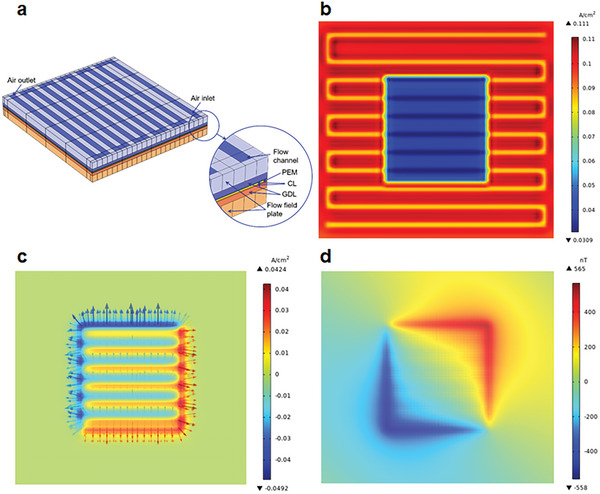
Current distributions at MEA surface due to PEMFC degradation heterogeneity. a) PEMFC model; b) PEMFC internal current distribution; c) PEMFC in‐plane current distribution; d) PEMFC magnetic field distribution due to in‐plane current. The distributions in Figure 2c,d are the difference between the state of local varied cathode exchange current density and the original state.

Numerical analysis is performed to clarify in‐plane current distribution due to PEMFC degradation heterogeneity. As illustrated in **Figure** **2**a, PEMFC model is situated between two single serpentine flow channels embedded in flow field plates, separated by gas diffusion layers (GDLs), catalyst layers (CLs), and PEM, model parameters are detailed in Table S1 (Supporting Information). Details of PEMFC model development and careful validation can be found in our previous work,^[^ ^23^ ^]^ while validation of PEMFC performance heterogeneity induced in‐plane current distribution can be seen in our previous work.^[^ ^22^ ^]^ To simulate degradation heterogeneity, cathode exchange current density of the selected area (middle zone of MEA surface) is reduced to half of the original value.

Figure 2b depicts internal current distribution among MEA, where the distinctive lower current density is evident in selected area. Furthermore, MEA surface exhibits a flow channel‐shaped pattern, where reactant concentration below the flow channel is different from those below the rib, aggravating uneven current distribution. Figure 2c further displays in‐plane current distribution, clearly indicating that due to uneven potential across MEA surface, in‐plane current flows (represented by arrows). More specifically, at the edge of selected area, due to larger potential difference, in‐plane current is pronounced, flowing from regions with lower catalyst activity to those with higher catalyst activity. Moreover, in the selected area, variation in reactant concentration and uneven potentials beneath flow channel and rib also contributes to in‐plane current. With Biot‐Savart law, in‐plane current will generate external magnetic field, as depicted in Figure 2d. However, to accurately sense magnetic field distribution around PEMFC (magnetic field variation due to in‐plane current is less than 1000 nT herein), multiple high‐resolution magnetic sensors are required.

### 2.2 Magnetic Array Imaging Device

From the literatures, two typical layout schemes for sensing PEMFC magnetic field have been proposed, as depicted in Figure S1 (Supporting Information). The 1st scheme inserts a magnetic sensor probe through the cooling hole of PEMFC stack,^[^ ^20^ ^]^ and the 2nd scheme places 30 magnetic sensors around PEMFC.^[^ ^24^ ^]^ Both schemes fail to measure magnetic field distribution among MEA surface, and thus cannot be utilized for clarifying PEMFC degradation heterogeneity. The main difficulty lies in that area of MEA surface is usually limited (25 cm^2^ in this work), integrating multiple magnetic sensors while minimizing mutual interference is challenging.

**Figure 3:**
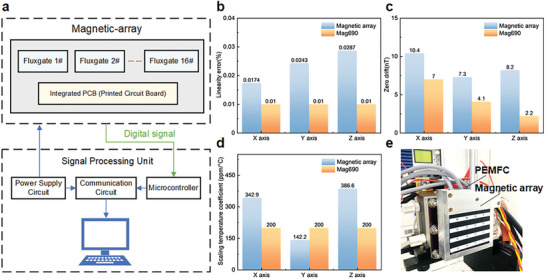
Schematics of designed magnetic array system and comparison results of sensor indexes. a) Innovative working principle of magnetic array system; b) Linearity error; c) Zero drift; d) Scaling temperature coefficient; e) Working diagram of magnetic array. Magnetic array indexes in Figure 3b–d are the average value of 16 fluxgates.

In this study, a magnetic array is designed to sense magnetic field distribution among MEA surface, as shown in Figure S1C (Supporting Information). As interference among analog signals from sensors in data transfer process can compromise measurement accuracy (depicted in Figure S2, Supporting Information), an innovative design is proposed by integrating PCB and sensors in magnetic array. As illustrated in **Figure** **3**a, magnetic array directly outputs digital signals to a microcontroller, thus mitigating interference between analog signals and enhancing immunity and reliability.

To assess sensing resolution of designed magnetic array, critical indexes such as linearity error, zero drift, and scaling temperature coefficient, are tested and compared with commercial three‐axis magnetic field sensor Mag690 (see Experimental Section). Figure 3b–d illustrates the comparison results, with raw data of 16 channels of the magnetic array available in Tables S2–S4 (Supporting Information). From comparisons, sensing resolution of designed magnetic array closely approaches that of Mag690, confirming the feasibility of visualizing PEMFC degradation heterogeneity. Notably, the designed magnetic array is placed on cathode bipolar plate side (Figure 3e), which can fully cover MEA surface (5 cm × 5 cm).

### 2.3 PEMFC Overall Performance Degradation

**Figure 4:**
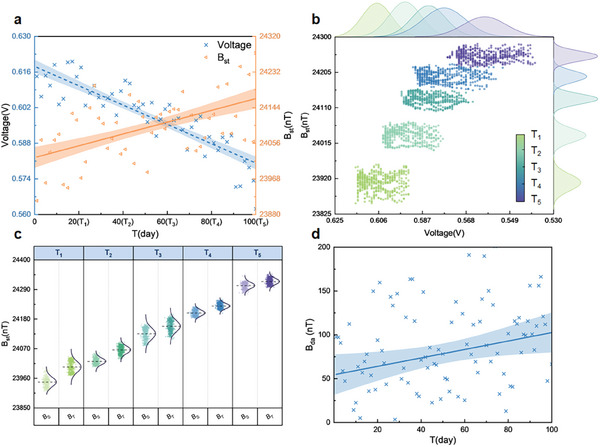
Voltage and magnetic field variations during AST. a) Voltage and magnetic field B_st_ variations within lifetime during AST. Voltage is the average value at the first 10 cycles per day; b) Correlation of voltage and magnetic field B_st_ at T_1_–T_5_. Each point represents voltage and averaged magnetic field of magnetic array during the first 10 cycles at T_1_–T_5_; c) Magnetic field B_S_ and B_T_ variations at T_1_–T_5_; d) Magnetic field B_da_ variation with lifetime during AST.

To investigate magnetic field variation during PEMFC degradation process, AST under startup–shutdown protocols is conducted, details can be found in Methods. As depicted in **Figure** **4**a,b, PEMFC voltage exhibits decreasing trend over its lifetime, indicating its declining performance. This performance drop is further validated with polarization curves and EIS results (see Experimental Section). Conversely, with PEMFC degradation, magnetic field during start cycles B_st_ (see Experimental Section) demonstrates an upward trend, as hindered redox reaction induces in‐plane current, exciting larger magnetic field. Moreover, five days at equal intervals during AST (marked as T_1_–T_5_) are collected and further illustrate this correlation of magnetic field and voltage in Figure 4b.

From degradation test, PEMFC performance fluctuations are universally observed, thus magnetic field during the first 10 cycles (B_S_) and the last 10 cycles (B_T_) on each of T_1_–T_5_ are demonstrated in Figure 4c, further confirming the increase of magnetic field with PEMFC daily operation. This can be attributed to Pt oxidation, as under startup–shutdown cycles, high voltage operation, and inadequate water management would make Pt oxidation, contributing to temporary loss of electrochemical active surface area (ECSA).^[^ ^25^ ^]^ Therefore, electrochemical reaction is constrained, which suppresses main current and intensifies in‐plane current, resulting in larger excited magnetic field. Figure 4d depicts the daily magnetic field variation B_da_ (the difference between B_T_ and B_S_ per day) during AST, where B_da_ exhibits an upward trend, indicating magnetic field variation due to daily performance loss increases during AST. As aforementioned, Pt oxidation is the main cause of daily performance losses, and therefore, Pt oxidation is more likely to occur as the experiment progresses.^[^ ^26^ ^]^

### 2.4 PEMFC Degradation Heterogeneity

In this section, in‐plane degradation heterogeneity within PEMFC is illustrated by analyzing magnetic field B_st_ distribution from designed magnetic array. To identify the main pattern of magnetic field, principal component analysis (PCA) is used to analyze magnetic field distribution (refer to Experimental Section for details). After PCA, the 2nd principal component is selected for the analysis, as the 1st principal component represents magnetic field excited by external current at bipolar plate, which remains relatively constant during PEMFC degradation.^[^ ^23^ ^]^

**Figure 5:**
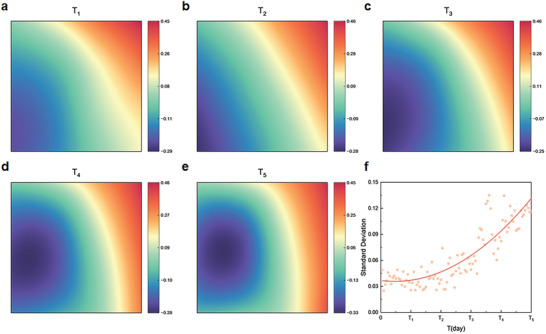
Principal component analysis of magnetic field distributions. a–e) Principal component analysis of magnetic field distributions at T_1_–T_5_; f Standard deviation variation of magnetic field distribution during AST.

According to magnetic array layout, the 2nd component is transformed into the matrix to illustrate its variation during PEMFC degradation, as shown in **Figure** **5**, where substantial variations are observed in the bottom right zone (hydrogen outlet) and the bottom left zones (air outlet) during AST. As aforementioned, the majority of internal current within MEA is main current, which does not interfere with current flow in other zones of MEA, resulting in similar magnetic field variations across all channels. However, as PEMFC degrades, more in‐plane current is induced due to degradation heterogeneity, leading to decreased synchronization between magnetic fields from different channels. Significant alterations in magnetic field patterns at hydrogen and air outlet zones suggest that substantial in‐plane current movement during degradation in these zones, contributing to increased degradation heterogeneity, this is consistent with previous studies regarding degradation mechanism under startup‐shutdown cycles (see Methods for more details).^[^ ^18^, ^27^, ^28^ ^]^ Moreover, from Figure 5a–e, difference among distinctive zones becomes more obvious, thus standard deviation, which can characterize the dispersion degree within magnetic field distribution, is used to quantify the degree of degradation heterogeneity due to in‐plane current during PEMFC degradation. As depicted in Figure 5f, it can be seen that standard deviation increases during AST, and the uptrend is more pronounced in the later stages, confirming that degradation heterogeneity is accelerated at the later stage, due to MEA structural collapse at hydrogen and air outlet.

### 2.5 PEMFC Material Characterization

**Figure 6:**
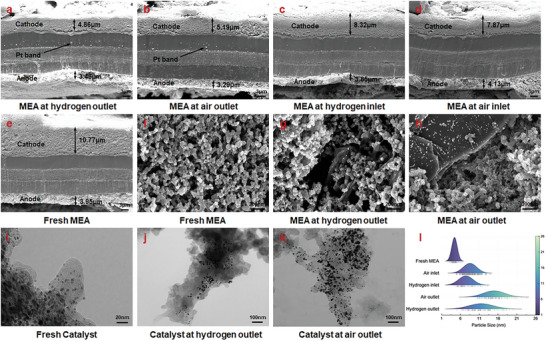
SEM and TEM images of catalyst layers and Pt catalysts at cathode. a–d) Cross‐sectional images of MEA after durability; e) Cross‐sectional image of fresh MEA; f Cathode catalyst layer of fresh MEA; g,h) Cathode catalyst layers of MEA after durability; i) Pt catalysts of fresh MEA; j,k) Pt catalysts of MEA after durability; l Pt particle size distributions of different MEA zones.

After the durability test, ex situ material characterization is performed, where SEM and TEM are used to probe micro and nano‐scale of cathode catalyst layer with special focus on Pt particles and carbon support, as illustrated in **Figure** **6**. Cross‐sectional images of MEA (Figure 6a–e) first validate the results from magnetic array analysis. The noticeable thickness reductions in cathode catalyst layer are more pronounced than anode catalyst layer. Among various zones, hydrogen outlet experiences the severest degradation, followed by that at air outlet. Compared with fresh MEA (Figure 6e), their thickness at hydrogen and air outlet are reduced to approximately half, indicating drastic carbon corrosion. Moreover, Pt particles dissolve from cathode catalyst layer and redeposit in membrane, forming band‐like Pt precipitation near cathode/membrane interface in Figure 6a,b.

Further in‐depth analysis of degradation behaviors at hydrogen and air outlet is performed, catalyst layer surfaces and Pt catalysts are investigated. For fresh MEA (Figure 6e), Pt particles are uniformly distributed on carbon support (Figure 6f,i), while after AST, severe carbon corrosion due to high electrode potential under startup–shutdown conditions significantly changes the microstructure at hydrogen outlet, leading to Pt particles detachment and agglomeration shown in Figure 6g,h captures more diverse degradation behaviors at air outlet, where Pt particles’ growth, migration, and carbon corrosion are simultaneously observed. Due to carbon corrosion, membrane is exposed and agglomerate Pt particles migrate and precipitate at membrane surface. Moreover, in Figure 6j, severe detachment of Pt particles from the edge of carbon support is observed at hydrogen outlet, with residual attached Pt particles agglomerated at the middle area. It can be caused by drastic carbon corrosion due to reverse current decay mechanism occurred at hydrogen outlet.^[^ ^29^ ^]^ As for Pt particles at air outlet shown in Figure 6k, it undergoes significant particle growth due to Pt dissolution and re‐deposition as Ostwald ripening, Pt coalescence, and agglomeration triggered by carbon corrosion.^[^ ^30^ ^]^

Furthermore, as depicted in Figure 6l, Pt particle sizes before and after AST are evaluated, and average particle sizes of fresh MEA and degraded MEA of air inlet, hydrogen inlet, air outlet, and hydrogen outlet are 4.43, 8.64, 7.44, 14.93, and 11.51 nm, respectively. The largest Pt particle size is found at air outlet, growing by 10.5 nm compared with fresh MEA, followed by hydrogen outlet with increase of 7.08 nm after AST. Such distributed and considerably increased Pt particle size after AST is caused by significant coalescence and Ostwald ripening, which is consistent with previous results in Figure 6. In summary, in‐plane degradation heterogeneity within MEA is confirmed by material characterization, and outlet zones suffer more severe degradation than inlet zones.

## 3 Conclusion

In this work, a magnetic array imaging device is designed to visualize PEMFC degradation heterogeneity. Compared with existing PEMFC degradation heterogeneity techniques, the designed magnetic array offers distinctive advantages, 1) the designed magnetic array is easily‐implemented and non‐destructive; 2) tested specified structure and material modification is not required for PEMFC.

Furthermore, to facilitate the application of magnetic array for PEMFC with larger MEA sizes, a three‐axis electric displacement platform can be equipped with the array, which can scan MEA surface following pre‐defined routes. Moreover, as the essence of magnetic imaging lies in sensing external magnetic field variation due to change in internal current, the designed magnetic array is not limited to PEMFCs, but is equally effective for other types of batteries such as lithium‐ion batteries, etc., providing insights into structure disintegration and material fracture.

In our future work, magnetic array imaging device will sense the external magnetic field of commercial PEMFC stacks to monitor its degradation heterogeneity. Also, artificial intelligence algorithm^[^ ^31^, ^32^ ^]^ will be applied in the analysis of magnetic field data to provide robust and explainable diagnostics.^[^ ^33^, ^34^ ^]^ Moreover, long‐term outdoor operation of fuel cell vehicles results in magnetic impurities adhering to PEMFC system, where the impact of these impurities on magnetic field detection will be further verified in real‐world experiment.

## 4 Experimental Section

### Experiments for Verifying Magnetic Array Sensing Resolution

Physical illustrations of the designed magnetic array and Mag690 (Bartington Instruments) can be seen in Figure S3 (Supporting Information), with technical parameters of Mag690 provided in Table S5 (Supporting Information). Experimental setups for verifying sensing resolution of magnetic array are depicted in Figure S4 (Supporting Information). Figure S4a (Supporting Information) displays a magnetic shielding cylinder to isolate external ambient magnetic field and magnetic field generation system. The magnetic field generation system controls a three‐axis Helmholtz coil energized to generate various stable currents at −700–+700 mA. According to Biot–Savart law, a stable current can generate constant magnetic field. Inside magnetic shielding cylinder, Figure S4b (Supporting Information) shows a three‐axis Helmholtz coil capable of creating a uniform magnetic field near the midpoint of its common axis, with the magnetic array positioned at the center of Helmholtz coil. Therefore, different static magnetic fields can be sensed by magnetic array and calibrated Mag690 to calculate linearity error, and both magnetic array and Mag690 operate for 12 h to calculate zero drift. Measurement results and technical parameters of Mag690 are considered as the benchmarks for sensor indexes. Moreover, as depicted in Figure S4c,d (Supporting Information), magnetic array is placed in the incubator to measure scaling temperature coefficient.

### In Situ Experiments

Detailed experimental operation and MEA parameters can be found in Table S6 (Supporting Information). Operation control of tested PEMFC (Yangtze Energy Technology) is implemented by a fuel cell testing system (Hephas Energy, HS‐150). AST under startup–shutdown conditions is carried out with the following steps: i) PEMFC is set to open‐circuit voltage (OCV) state; ii) startup–shutdown cycles of which currents vary from 0 and 25 A, and each current is held for 60 s; iii) above steps are repeated for 360 cycles per day. Current and voltage curves during startup–shutdown cycles can be seen in Figure S5 (Supporting Information). During AST, polarization curves and EIS are interval sampled every 2400 startup–shutdown cycles to characterize PEMFC performance. PEMFC is run for 100 days in AST. A magnetic array with a resolution of 0.1 nT is placed outside cathode bipolar plate to sense magnetic field excited by in‐plane current in the whole lifecycle, and vertical distance between magnetic array and cathode bipolar plate surface of tested PEMFC is 5 mm.

### Ex Situ Experiments

Once in situ experiments are finished, MEA of tested PEMFC is taken apart for ex situ analysis, and its air inlet and outlet, hydrogen inlet, and outlet zones are sampled. MEA cross‐section and surface morphology of cathode catalyst layer are investigated by SEM (Genimi SEM 500, Carl Zeiss Microscopy Ltd.). Degradation behaviors of Pt catalyst are studied with TEM (JEM‐2011, Japan Electronics Co., Ltd). Moreover, a fresh sample is taken from an unused MEA for comparison.

### Data Analysis

Magnetic array can detect magnetic field of three orthogonal components. As depicted in Figure S1 (Supporting Information), X direction was perpendicular to MEA surface and magnetic field in X direction was excited by in‐plane current. Therefore, when PEMFC operated during every startup cycles, the average magnetic field of magnetic array in X direction was collected for analysis, marked as B_st_. During AST, 20, 40, 60, 80, and 100 days are denoted as T_1_, T_2_, T_3_, T_4_ and T_5_ for analyzing the degradation process. Magnetic field variation within daily operation was marked as B_da_, which can be calculated by:

(1) $$B_{\mathrm{da}}=B_{T}-B_{S}$$

B_S_ and B_T_ denote the average magnetic field of magnetic array during the first 10 startup cycles and the last 10 startup cycles in a day.

### Polarization Curves and EIS Results

**Figure 7:**
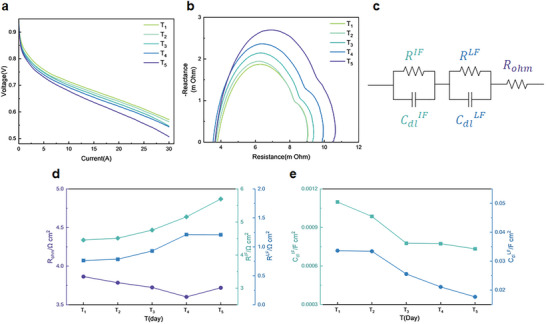
Polarization curve and EIS results. a) Polarization curves during startup–shutdown cycles; b) EIS during startup–shutdown cycles; c) Equivalent circuit for EIS analysis; d,e) Variations of equivalent circuit elements.

PEMFC degradation evaluated by polarization curves is depicted in **Figure** **7**a. PEMFC performance gradually decreased during AST and its degradation was accelerated at T_4_‐T_5_ stage. Corrosion of carbon support severely degrades electrode structure, so that porous electrode would lose its structural integrity, which significantly hinders mass transfer and results in a significant performance loss.

As for EIS results, Figure 7b depicts that middle‐frequency and low‐frequency impedance significantly increases, while little variation is observed in high‐frequency impedance. It indicates that ohmic resistance slightly decreases, and performance degradation is mainly caused by catalyst layer degradation. To better describe impedance characteristics during PEMFC degradation, an equivalent circuit is applied for EIS analysis. Figure 7c consists of a serial connection of the following elements: i) an ohmic resistance $\left(R^{\Omega }\right)$ is mainly dominated by membrane resistance; ii) a cathode charge transfer resistance (*R^IF^*) is in parallel with a constant phase element *C_dl_* ^*IF*^ for distributed double‐layer capacitive effects, which both contribute to cathode activation loss;^[^ ^35^ ^]^ iii) *R^LF^* and *C_dl_* ^*LF*^ are catalyst layer resistance and capacitance at cathode for mass transport limitation dominated by diffusion processes.^[^ ^36^ ^]^ From Figure 7d,e, an increase in *R^IF^* along with a decrease in *C_dl_* ^*IF*^, exactly correspond to ECSA loss, which is explained by decrease in surface area of Pt catalysts and carbon support. Moreover, ECSA loss accompanies morphological changes in catalyst layer, such as Pt dissolution and agglomeration, which correlates with mass transport process.^[^ ^37^ ^]^ Due to Pt dissolution, the rise of *R^LF^* is attributed to increased resistance in air diffusion within catalyst layer. *C_dl_* ^*LF*^ represents the quantity of stored air within polymer, which consequently decreases with degradation caused by Pt dissolution. Additionally, reduction of ohmic resistance is in agreement with Castanheira studies,^[^ ^8^ ^]^ suggesting an increase in surface hydrophilicity during carbon corrosion, which may retain more water within electrode instead of evacuating it through gas diffusion layer. Moreover, more Pt catalysts have dissolved from cathode catalyst layer and re‐deposited in membrane (Figure 6a,b) could also result in reduction of ohmic resistance.

### Reverse Current Decay Mechanism

**Figure 8:**
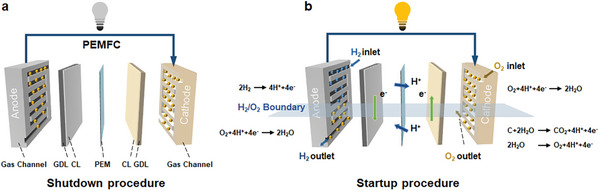
Mechanism of degradation heterogeneity under startup‐shutdown conditions. a) Shutdown procedure; b) Startup procedure.

Reverse current decay mechanism is widely acknowledged as the fundamental cause for PEMFC degradation under startup‐shutdown conditions.^[^ ^38^ ^]^ As illustrated in **Figure** **8**a, before PEMFC startup, both channels are filled with air. With hydrogen supplied into anode, hydrogen, and air coexist on anode side, forming a hydrogen–air interface (Figure 8b), where anode side is separated into two different reaction regions, that is, hydrogen‐rich region with hydrogen oxidation, and air‐rich region with oxygen reduction. Therefore, electrons and protons are required for oxygen reduction reaction, where electrons are supplied from hydrogen‐rich region, while to lower conduction resistance, protons will be obtained by crossing PEM from cathode to anode. This process involves protons supply through carbon support corrosion and oxygen evolution reaction, leading to cathode degradation. Furthermore, hydrogen was injected from inlet, leading to abundant air in outlet zone. Therefore, carbon corrosion predominantly occured in the zone near hydrogen and air outlets at cathode. From Figure 8, it is evident that various internal reactions that occur in these zones necessitate the movement of protons and electrons, from which in‐plane current is generated. Furthermore, carbon corrosion exacerbates damages to microstructure and hydrophobicity in catalytic layer, rendering it more susceptible to flooding and subsequently increasing mass transport resistance.^[^ ^39^ ^]^ In this scenario, more catalyst sites were submerged in water and made inaccessible to reactants. Therefore, limited electrochemical reaction, induced by flooding at outlet zone, further facilitated in‐plane current. During PEMFC lifetime, the difference in electrochemical reaction efficiencies between inlet and outlet regions continues to widen, which promoted in‐plane current movement, and accelerated PEMFC degradation. Therefore, with aforementioned analysis, the coupling of carbon corrosion and flooding, as well as their non‐uniformity distribution (prominent at hydrogen and air outlet), amplified in‐plane degradation heterogeneity within PEMFC.

### Statistical Analysis

PCA is a widely used dimension reduction technique for recognizing major patterns within a large dataset. To identify major variation patterns within magnetic field as PEMFC degradation, B_st_ is extracted. Let's denote *i^th^* data frame at *j^th^* startup–shutdown cycle as $b_{i}^{j}={\left[b_{i,1}^{j},\text{…},b_{i,16}^{j}\right]}^{⊤}$. Suppose there are *m* data frames within each cycle, i.e., *i* = 1, ⋅⋅⋅, *m*, and *n* cycles in each day, that is, *j* = 1, ⋅⋅⋅, *n*. Since the magnetic field at the end of a cycle is significantly different from the begin of its next cycle, mean magnetic field within each cycle was removed from original data $b_{i}^{j}$, and obtained data are denoted as ${\tilde{b}}_{i}^{j}$. After that, a data matrix M was obtained by putting each data frame as a row vector, i.e., $M={\left[{\tilde{b}}_{1}^{1},\text{…},{\tilde{b}}_{m}^{1},\text{…},{\tilde{b}}_{i}^{j},\text{…},{\tilde{b}}_{1}^{n}\cdots ,{\tilde{b}}_{m}^{n}\right]}^{⊤}$. Then, MATLAB function “pca” was adopted to extract principal components, that is, major variation patterns, from data matrix.

## Conflict of Interest

The authors declare no conflict of interest.

## Supporting information

Supporting Information

## Data Availability

The data that support the findings of this study are available from the corresponding author upon reasonable request.
