# Supplementary material for: Magnetic Array‐Aided Visualizing PEMFC Degradation Heterogeneity
Source: Adv Sci (Weinh). 2024 Jun 17;11(31):2403631. doi: 10.1002/advs.202403631 (PMC11336923; doi:10.1002/advs.202403631)
Supplement: Supplementary file 1 — Supporting Information [file ADVS-11-2403631-s001.docx]

Supporting Information

Magnetic Array-aided Visualizing PEMFC Degradation Heterogeneity

Yuning Sun, Lei Mao*, Zhiyong Hu, Xiaoyu Zhang, Ranran Peng


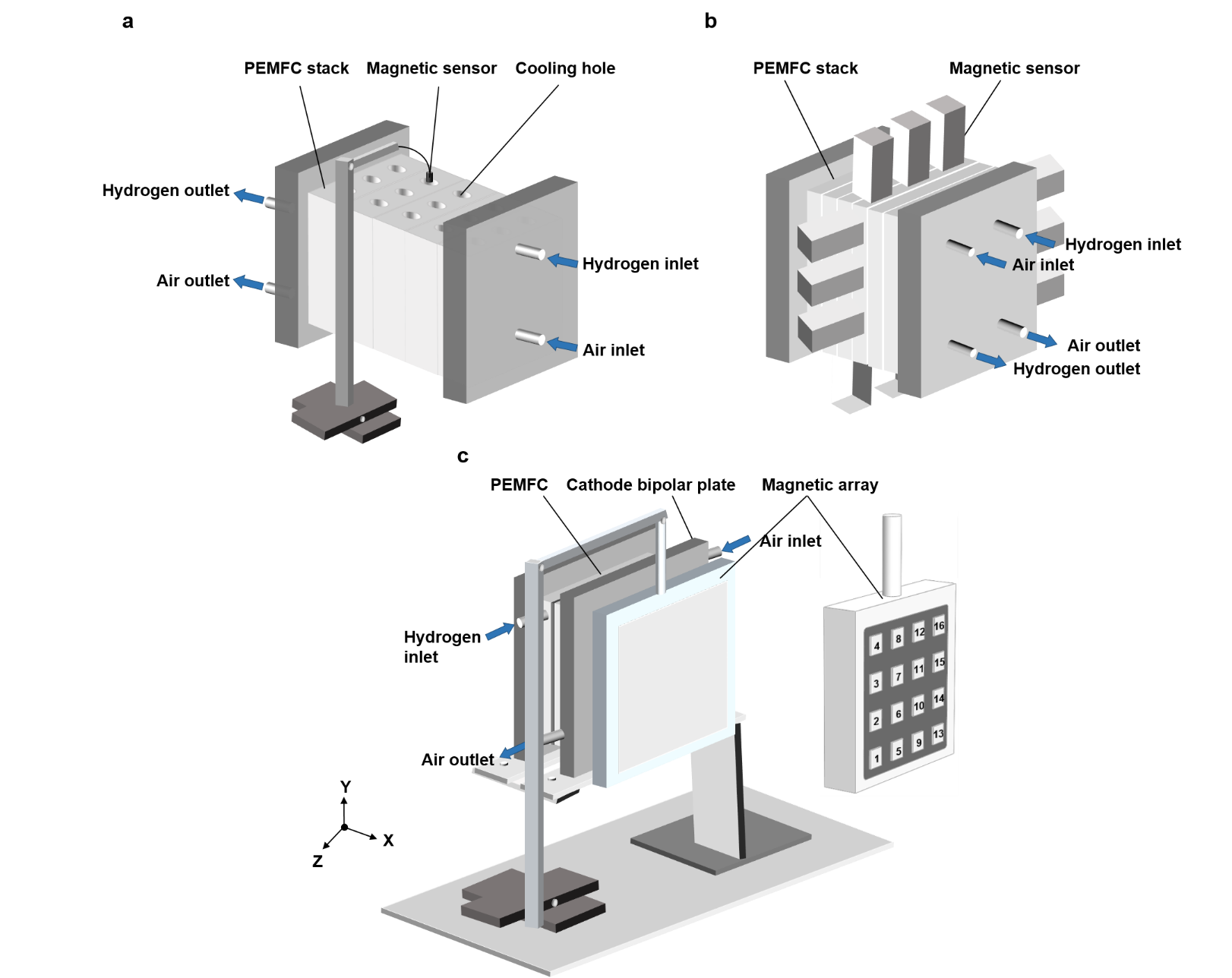


Figure S1. Experimental equipment of magnetic field in previous studies and our study. a In referred study^20^, magnetic field measured by inserted a magnetic sensor into cooling holes of PEMFC stack; b in referred study^24^, magnetic field measured by an array of magnetic sensor around PEMFC stack; c Magnetic array used in our measurement.


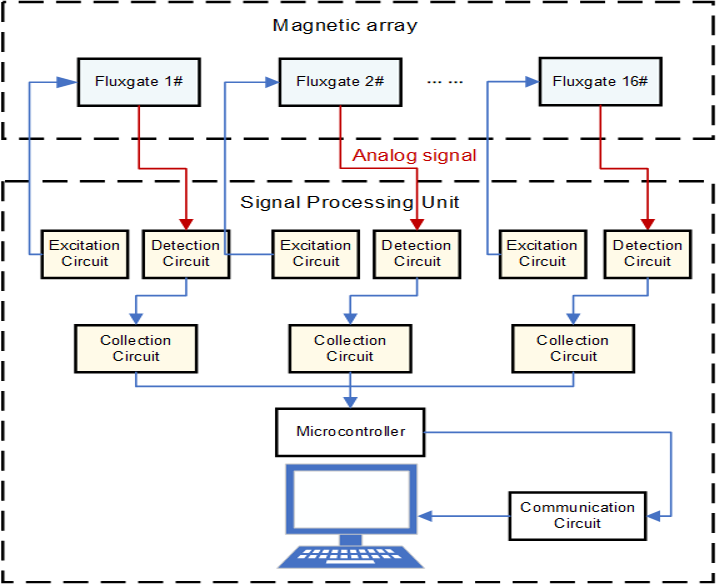


Figure S2. Conventional working principle of magnetic array system.


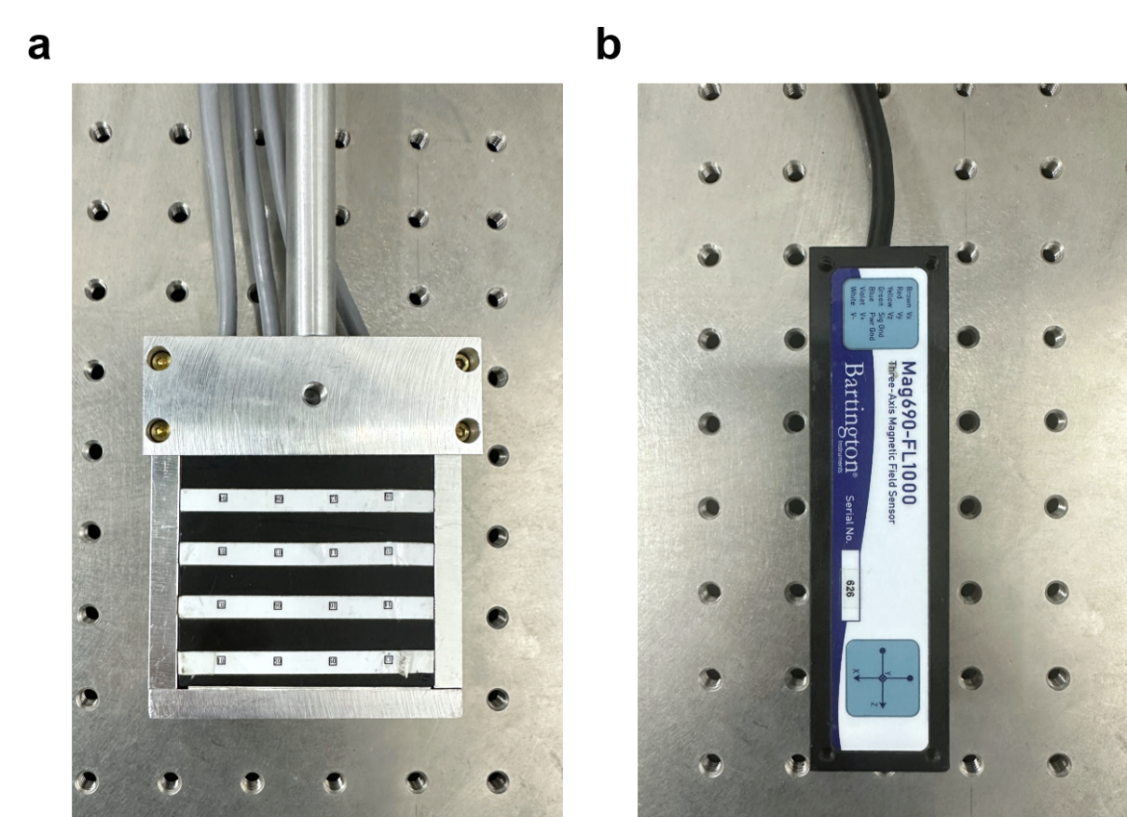


Figure S3. Physical illustrations of magnetic array and Mag690. a Magnetic array; b Mag690.


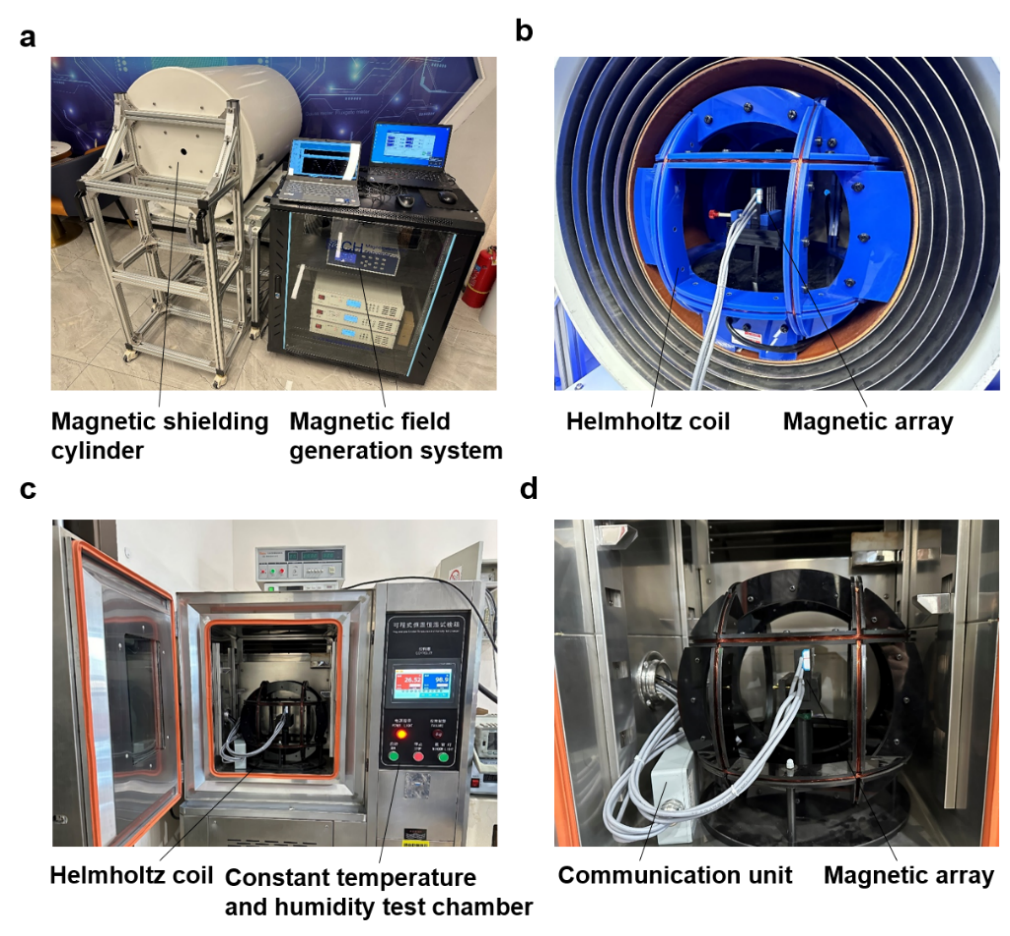


Figure S4. Experimental setups for verifying the sensing resolution of magnetic array. a Magnetic shielding cylinder and magnetic field generation system; b Magnetic array placed inside the three-axis Helmholtz coil in magnetic shielding cylinder; c Constant temperature and humidity test chamber; d Magnetic array placed inside the three-axis Helmholtz coil in the incubator.


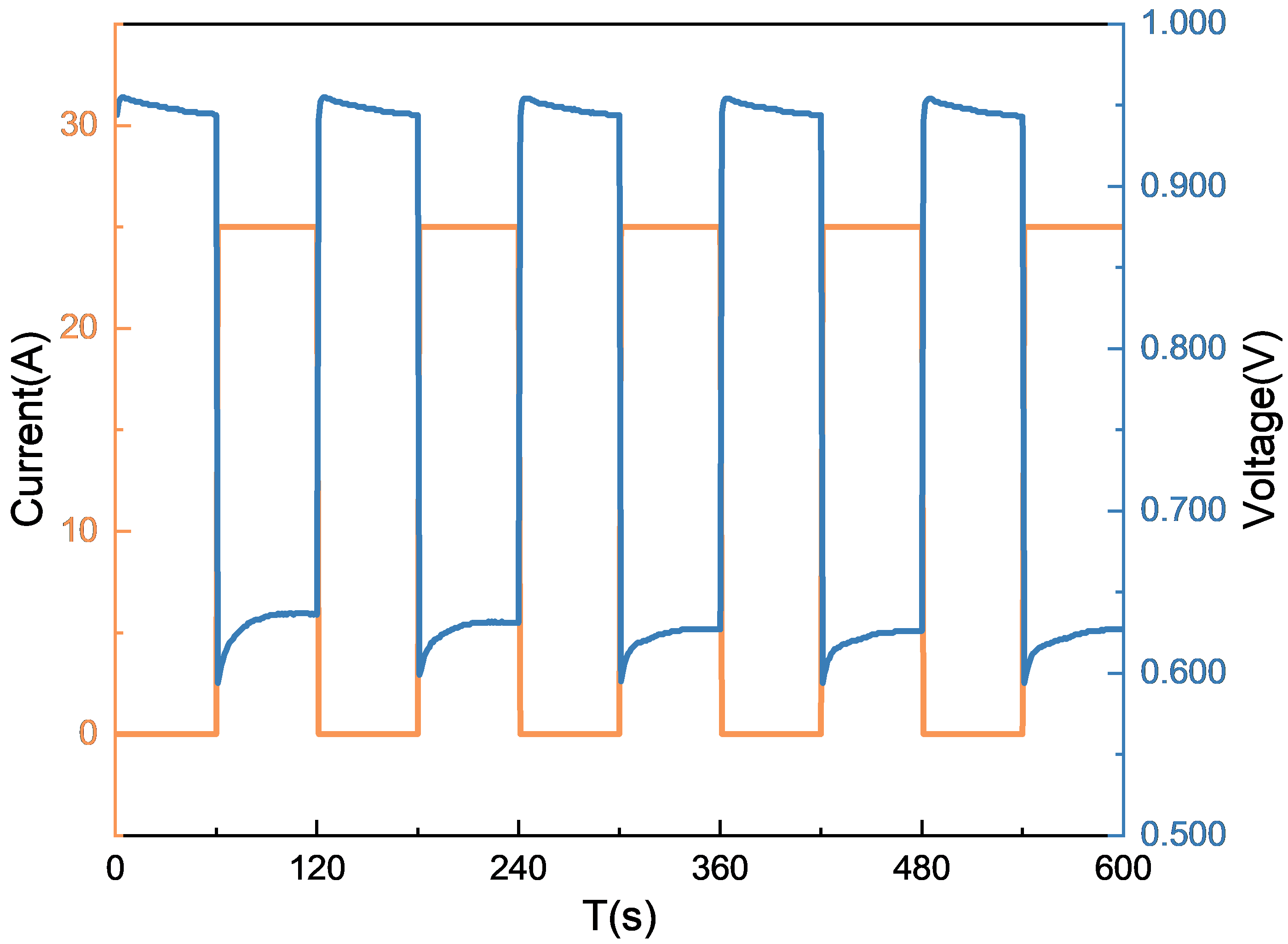


Figure S5. Startup–shutdown cycles during AST.

**Table S1.** Operation parameters used in PEMFC model.

| Parameters | Value |
| --- | --- |
| PEMFC temperature (K) | 323.15 |
| Reference pressure (atm) | 1 |
| Electric conductivity of PEM (S/m) | 9.825 |
| Electric conductivity of GDL (S/m) | 222 |
| CL porosity | 0.3 |
| GDL porosity | 0.4 |
| CL permeability (m^2^) | 2.35×10^-12^ |
| GDL permeability (m^2^) | 1.18×10^-11^ |
| Cathode transfer coefficient | 1 |
| Specific surface area (1/m) | 1×10^7^ |
| Boundary electric potential (V) | 0.9 |
| Mass fraction of oxygen | 0.20 |
| Mass fraction of hydrogen | 0.96 |
| Inlet water molar fraction | 3.732×10^-2^ |
| Inlet hydrogen molar fraction | 0.963 |
| Inlet oxygen molar fraction | 0.202 |
| Inlet nitrogen molar fraction | 0.761 |
| Anode exchange current density (A/m^2^) | 100 |
| Cathode exchange current density (A/m^2^) | 0.001 |
| Anode stoichiometry | 2 |
| Cathode stoichiometry | 3.5 |
| Anode inlet velocity (m/s) | 1.16 |
| Cathode inlet velocity (m/s) | 4.83 |

**Table S2.** Linearity error of magnetic array.

| Channels | X axis(%) | Y axis(%) | Z axis(%) |
| --- | --- | --- | --- |
| 1 | -0.0074 | 0.0352 | 0.0349 |
| 2 | 0.0048 | 0.0325 | 0.0357 |
| 3 | -0.0138 | 0.0292 | 0.0294 |
| 4 | 0.0256 | 0.0196 | 0.0183 |
| 5 | 0.0291 | 0.0058 | 0.0081 |
| 6 | 0.0063 | 0.0117 | 0.0150 |
| 7 | 0.0293 | 0.0198 | 0.0290 |
| 8 | 0.0219 | 0.0172 | 0.0230 |
| 9 | -0.0060 | 0.0401 | 0.0439 |
| 10 | 0.0505 | 0.0152 | 0.0243 |
| 11 | 0.0666 | 0.0132 | 0.0211 |
| 12 | 0.0085 | 0.0279 | 0.0275 |
| 13 | 0.0322 | 0.0260 | 0.0330 |
| 14 | 0.0179 | 0.0334 | 0.0391 |
| 15 | 0.0130 | 0.0300 | 0.0405 |
| 16 | 0.0002 | 0.0318 | 0.0364 |
| Average value | 0.0174 | 0.0243 | 0.0287 |

**Table S3.** Zero drifts of magnetic array and Mag690 in 12 hours.

| Channels | X axis(nT) | Y axis(nT) | Z axis(nT) |
| --- | --- | --- | --- |
| 1 | 9.9 | 5.7 | 5.7 |
| 2 | 18.2 | 9 | 9.4 |
| 3 | 7.3 | 5 | 4.7 |
| 4 | 5.9 | 7.1 | 5.9 |
| 5 | 8 | 12.3 | 13.3 |
| 6 | 13.5 | 4.4 | 8.5 |
| 7 | 7.8 | 5.2 | 6.4 |
| 8 | 7.7 | 8 | 9.6 |
| 9 | 6.5 | 7.7 | 8.9 |
| 10 | 5 | 8 | 5.9 |
| 11 | 13.6 | 6.7 | 10.2 |
| 12 | 7.2 | 7.5 | 10.5 |
| 13 | 9.7 | 11.3 | 9.5 |
| 14 | 20.4 | 7.9 | 8.8 |
| 15 | 14.4 | 5.4 | 6.2 |
| 16 | 12 | 5.5 | 7.3 |
| Average value | 10.4 | 7.3 | 8.2 |
| Mag690 | 7 | 4.1 | 2.2 |

**Table S4.** Scaling temperature coefficient of magnetic array.

| Channels | X axis(ppm/°C) | Y axis(ppm/°C) | Z axis(ppm/°C) |
| --- | --- | --- | --- |
| 1 | 127.7 | -41.6 | 551.2 |
| 2 | 289.8 | 68.4 | 506.1 |
| 3 | 151.1 | 289.2 | 532.8 |
| 4 | 218.2 | 428.4 | 581.9 |
| 5 | 206.6 | -62.9 | 217.8 |
| 6 | 366.7 | -52.8 | 201.4 |
| 7 | 306.8 | 168.6 | 345.6 |
| 8 | 426.9 | 261.9 | 365.2 |
| 9 | 541.1 | -95.1 | 148.6 |
| 10 | 482.5 | 72.5 | 180.8 |
| 11 | 570.2 | 240.3 | 408.2 |
| 12 | 588.0 | 426.2 | 451.1 |
| 13 | 193.0 | -21.6 | 404.7 |
| 14 | 217.5 | 208.4 | 565.3 |
| 15 | 568.4 | 117.0 | 383.0 |
| 16 | 231.9 | 268.5 | 341.4 |
| Average value | 342.9 | 142.2 | 386.6 |

**Table S5.** Technical parameters of Mag690.

| Parameters | Value |
| --- | --- |
| Measurement span | ±1000 μT |
| Operation temperature | -40 ℃ ~ 55 ℃ |
| Resolution | 0.1 nT |
| Linearity error | 0.01% |
| Start/stabilize time | 0.5s |
| Magnetic hysteresis | <0.1% Measuring span |
| Scaling temperature coefficient | ±200 ppm/°C |

**Table S6.** Operation parameters and MEA parameters of tested PEMFC.

| Parameters | PEMFC |
| --- | --- |
| Number of cells | 1 |
| Thickness of membrane,$\mu m$ | 100 |
| Surface area, cm^2^ | 25 |
| Loading of platinum, mg/cm^2^ | 0.15 @ anode; 0.35 @ cathode; |
| Flow channel | Serpentine |
| PEMFC temperature, ℃ | 50 |
| Anode/cathode stoichiometric | 1.5/3.5 |
| Relative humidity | 75% |
